# The value of regulating stocking densities in aquaculture must not be dismissed: a reply to Saraiva et al. 2022

**DOI:** 10.3389/fvets.2023.1335667

**Published:** 2024-01-03

**Authors:** Helen Lambert, Amelia Cornish, Doug Waley

**Affiliations:** ^1^Animal Welfare Consultancy, Kingsteignton, United Kingdom; ^2^Eurogroup for Animals, Brussels, Belgium

**Keywords:** aquaculture, stocking density, legislation, fish, policy, regulation, welfare

## 1 Introduction

We would like to rebut the argument made by Saraiva et al. (1) in their paper “Finding the “golden stocking density”: a balance between fish welfare and farmers' perspectives,” that stocking densities should not be regulated in aquaculture. In their paper, the authors make the case that although inappropriate stocking densities in aquaculture can negatively impact fish welfare, they should not be regulated for the following reasons. First, they state that stakeholders are searching for an “optimal stocking density” and that this outlook is biased from the outset. Second, they argue that conclusions regarding stocking density vary from study to study. Third, they argue that there is no functional meaning or biological need behind stocking density. Fourth, they argue that because stocking density interacts with many other welfare parameters and indicators, it should only be used as a farmer management tool, as its regulation would be unworkable and ineffective. In this paper, we dispute these arguments and provide evidence to show that stocking density can, and is already, reliably and effectively used in aquaculture, including to govern welfare and, along with other key indicators, offers a species-relevant meta-indicator and intervention tool.

## 2 Few people are looking for a “golden stocking density”

In their mini review, Saraiva et al. (1) claim that “From fish farmers to certifiers and even animal advocacy groups, all parties…[are] searching for the “golden stocking density” (p2) and that this search is biased depending on the stakeholder's agenda. There are, of course, many perspectives to consider here, but from an advocacy perspective, advocates are not seeking regulation of a “golden stocking density” but are rather seeking stocking density limits that reduce many welfare risks, including chronic welfare issues, and that allow for a good life for farmed fish (2).

Both advocates and certifiers are drawing upon the extensive experience with terrestrial species in agriculture, which has repeatedly shown the importance and value of stocking density in welfare regulations (3–6) (also see Section 5). This is a legitimate and important approach as the multifactorial relationships between stocking density and animal welfare in aquaculture, as defined by fish farmers and expert groups (7), reflect very closely the relationships between stocking density and animal welfare in terrestrial agriculture (8, 9). Although with the additional impacts that stocking densities have upon water quality in aquaculture, the consequences are potentially worse.

Academic works have sought optimal stocking densities and identified many reasons why it is difficult to determine one for a fish species in aquaculture (10). Whilst we agree that it is difficult to set “optimal” stocking density levels, when done correctly, stocking density limits are used to protect animal welfare, preventing issues before they develop. In fact, operational welfare input indicators, like stocking density, are often considered valuable preventative tools (11–13) and are typically the ideal practical input measure for legislation (14–16).

## 3 Different studies will paint different pictures

Saraiva et al. (1) list several examples of studies (p2) where the outcomes regarding optimal stocking densities differ while trending toward poor outcomes toward higher and sometimes lower stocking densities. Upon close inspection, these studies often differ in experimental design, including key factors such as fish age and variety in the limited number of indicators assessed, so it is no surprise that they produced different results. For instance, the authors list several studies on Atlantic salmon (*Salmo salar* L.) (17–19), each using different holding facilities, varying from sea cages to land-based tanks of differing sizes. Moreover, the comparisons included also vary significantly between very high and very low stocking densities, and such extremes are known to cause welfare issues (20). In fact, the studies listed by the authors corroborate our point that a moderate threshold can be successfully used to mitigate welfare issues and prevent fish from being farmed at densities that are too high or too low for their needs.

In addition, many studies extrapolate from small-scale experiments rather than commercial farms, resulting in significant issues (21). To achieve reliable and consistent results, study designs must be controlled to achieve reproducible results (18) and use a set of comprehensive indicators in terms of the Five Domains model (22). However, we do recognize the challenges involved with achieving replicability in aquaculture (17).

## 4 Stocking density is relevant to function, behavior, and mental states

In their paper, the authors (1) discuss the three approaches to animal welfare, namely, the feelings-based approach, which considers the animal's mental state; the function-based approach, which is concerned with the animal's health and functioning; and the nature-based approach, which refers to the need for a natural environment and the expression of natural behavior (23, 24). Then, they rightly say that integrating the three can help operationalise the animal welfare concept. However, what is then confusing and rather alarming is that they disregard stocking density as a welfare indicator because “there is no functional meaning or biological need behind these parameters” (p. 3). Our response to this statement falls under two main themes. First, we argue that stocking density is highly embedded in function, and health often relies on optimal stocking densities (25). For example, North et al. (26) found that fin condition deteriorates as densities increase in rainbow trout (*Oncorhynchus mykiss*), and fin erosion meant that fish held at 40 and 80 kg m^−3^ had significantly smaller fins than those held at 10 kg m^−3^. Furthermore, in another study, the onset of morbidity was faster, and the total morbidity was higher in Atlantic salmon kept at higher stocking densities and exposed to Amoebic gill disease, compared with those at lower densities (27). Similarly, reducing the stocking density in caged Nile tilapia (*Oreochromis niloticus*) also resulted in a reduced risk of disease outbreak, fewer mortalities and deformities, removing the need for prophylactic drugs, and resulting in improved growth performance due to a better feed conversion rate (28).

Our second argument concerning this statement is that it fails to consider the importance of the other two approaches, which Saraiva et al. said were significant when considering welfare. Fish have historically been devalued in terms of sentience (29–31), and we still know little about the importance and motivations of some of their natural behaviors and the psychological impact when they are thwarted (31–34). This does not mean they should be neglected, as mental states and behavior are core welfare components (30). Evidence also shows poor mental states can negatively affect physical health (34–36).

If we only address the functional aspects of an animal's environment by, for example, managing water quality, although the fish may be able to physically cope with high stocking densities, it is unclear what impact severe restriction on behavior may have on their mental wellbeing (33, 34, 37). This has been seen repeatedly in terrestrial-farmed species, where certain negative impacts of intensive production are mitigated or managed without addressing the issue's root. For example, beak-trimmed hens (Gallus gallus domesticus) may not be able to perform injurious pecking on conspecifics, but they are still frustrated and stressed by their impoverished environment (38). Therefore, not only is stocking density highly relevant in terms of animal functioning, but as high stocking densities can grossly impede natural behaviors in some species (39), the behavioral aspect of welfare is also impacted, which likely results in negative emotional states such as frustration and stress in the fish (3, 31, 34).

## 5 Stocking density as an effective tool for legislation

Saraiva et al. (1) argue that “Legislation directly limiting stocking density is likely to be unworkable…” (p. 4). We disagree as there are already relevant, proven track records for using stocking densities in aquatic- and terrestrial-farmed animal welfare legislation. For example, legislation protecting broiler chickens often states a maximum stocking density [e.g., the EU (40), Australia (41), and the UK (42)]. Similarly, stocking densities or space allowances are also commonly included for pigs [e.g., the EU (43), Australia (44), and the UK (42)].

Furthermore, stocking density regimes are already utilized in aquaculture legislation and certification schemes worldwide, with promising indications for more widespread use. For example, the Norwegian Aquaculture Act (2005) (45); the Chilean Aquaculture Regulation N^o^ 1503 (2013) (46); the EU legislation for organic aquaculture in Regulation (EU) 2018/848 (47); the UK RSPCA Assured scheme for salmon (48) and trout (49); and recently in Maine (USA), the Act L.D.1951; An Act Regarding Marine Finfish Aquaculture (50), all include stocking densities for aquaculture. And, as far as we know, there are no cases where a legislative regime or certification scheme has found the implementation of stocking limits to be problematic, causing it to remove or raise its stocking density limits.

In their mini-review, Saraiva et al. (1) suggested that in place of stocking density limits, “…a more practical option might be to prescribe acceptable levels of different welfare indicators (e.g., water quality, health, nutritional condition and behavioral indicators),….” A similar argument was made by the New Zealand (NZ) government when they decided against lowering broiler stocking densities, as they argued that animal welfare should be addressed more holistically, using a combination of other factors, including management skills and environmental issues. Sankoff (51) reviewed NZ's Animal Welfare Act 1999 and claimed this decision to be “…entirely unsatisfactory” (p. 23), and we agree. Sankoff (51) argues that such disregard for the role of stocking density within animal welfare prioritized “a more industry friendly standard in spite of the proven correlation between high stocking densities and poor animal health” (p.23).

Stocking density as a legislative tool provides clearly defined quantifiable welfare parameters that are easily implemented, delivering important meta-indicators and interventions for animal welfare. For example, the EU Directive 2007/43/EC outlines broiler stocking density limits and in the Netherlands, the Dutch Animals Act (2011) (40) requires farmers at higher stocking densities to monitor animal-based indicators, such as incidences of footpad dermatitis. Then, if scores are too high, farmers must create improvement plans, which often include lowering stocking density (13).

## 6 Consumers value stocking densities

Saraiva et al. (1) correctly contend that “Public concern about the welfare of farmed fish has been confirmed in surveys at European scale… and consumer pressure is responsible for enhanced control and regulation in welfare assurance schemes… and by policy advice or regulation at national and international/European level… Therefore, in some markets at least, there are premiums to be charged for fish cultured under welfare assurance schemes.”

Aquaculture is one of the fastest-growing animal food-producing sectors (52). Fish meat continues to grow in popularity, with many consumers perceiving it as a healthier alternative to other meats (53). Aquaculture has become increasingly intensive to meet the demand, giving rise to animal welfare concerns in the industry (52). Furthermore, intensive fish farming with high stocking rates has been the subject of television documentaries and newspaper articles around the world [for example (54) and (55)]. Negative representation in the media has also led to aquaculture losing consumer trust (56, 57).

Consumers appear to value the transparency that stocking density figures provide. For example, in Australia, a Humane Society International survey of 1,400 Australian consumers found that an overwhelming 98% of respondents wanted all “free range” egg cartons to display the outdoor stocking density for the hens (58).

Similar consumer sentiment can be found for fish welfare. For instance, in Germany, a choice experiment found that consumers showed a preference and an increased willingness to pay for organic labeled fish that provided information about animal welfare, including stocking density (52). In contrast, additional information about the environmental consequences had no effect (52).

## 7 Conclusion

In this paper, we have presented our counter-argument to that made by Saraiva et al. (1) in their paper “Finding the “golden stocking density”: a balance between fish welfare and farmers' perspectives”, which disregards the value of stocking densities in regulating aquaculture. We have provided clear evidence to show that stocking densities are already successfully being used in terrestrial farming and aquaculture and that they provide a valuable preventative measure for safeguarding animal welfare. We recognize that more research is needed in some areas to establish commercially relevant stocking densities for all aquaculture species. However, such research and efforts are needed to ensure that fish are kept in numbers that allow them to fulfill normal behaviors, maintain optimum health, and mitigate the risk of chronic stress. Given the growing pressure that the aquaculture industry is under to produce fish meat, it is vitally important that we recognize the value that key inputs such as stocking density have in addressing welfare issues.

## Author contributions

HL: Writing—original draft, Writing—review & editing. AC: Writing—original draft. DW: Conceptualization, Funding acquisition, Writing—review & editing.
